# Balancing religious obligations and cultural integration—female foreign Muslims’ healthcare experiences in Japan: a qualitative study

**DOI:** 10.1016/j.xagr.2025.100477

**Published:** 2025-03-12

**Authors:** Ayako Kohno, Maznah Dahlui, Inge Dhamanti, David Koh, Hanif Abdul Rahman, Takeo Nakayama

**Affiliations:** 1 Department of Health Informatics, School of Public Health, Graduate School of Medicine, Kyoto University, Kyoto, Japan (Kohno and Nakayama); 2 Department of Research Development and Innovation, University of Malaya Medical Centre, Kuala Lumpur, Malaysia (Dahlui); 3 Centre of Population Health, Faculty of Medicine, Universiti Malaya, Kuala Lumpur, Federal Territory of Kuala Lumpur, Malaysia (Dahlui); 4 Department of Health Policy and Administration, Faculty of Public Health, Universitas Airlangga, Surabaya, Indonesia (Dahlui and Dhamanti); 5 Center of Excellence for Patient Safety and Quality, Universitas Airlangga, Surabaya, Indonesia (Dhamanti); 6 Saw Swee Hock School of Public Health, Tahir Foundation Building, National University of Singapore, Singapore, Singapore (Koh); 7 Digital Public Health Graduate Programmes, PAPRSB Institute of Health Sciences, Universiti Brunei Darussalam, Gadong, Brunei Darussalam (Rahman)

**Keywords:** Healthcare experiences, Japan, Muslim, Qualitative study

## Abstract

**Background:**

When foreign Muslim women living in Japan seek healthcare, they may encounter some issues about their Islamic obligations. Their experiences are not well studied to date. This study aimed to describe the experiences of foreign Muslim women when receiving healthcare at clinics and hospitals in Japan.

**Materials and Methods:**

This is a qualitative study using in-depth interviews with 28 foreign Muslim women from Indonesia and Malaysia who were living in Japan at the time of the study. Data were transcribed and phenomenological analysis was performed to describe their lived experiences.

**Result:**

Three themes emerged: (1) meeting religious obligations as a female Muslim, (2) pregnancy and childbirth-related experiences, and (3) experiences of accessing healthcare in Japan.

**Conclusion:**

Foreign Muslim women were not sure how to balance their religious obligations and culturally integrate into the Japanese healthcare system by conveying their needs and wishes to Japanese healthcare providers. The findings of this study may be useful for Japanese healthcare providers when interacting with foreign Muslim patients.

## Introduction

Healthcare needs are bound by many factors related to culture and religion.^1^^,^^2^ When Muslim patients migrate to non-Muslim countries, the healthcare provided in respective destination countries may not be sufficient to satisfy their healthcare needs as Muslims.^3^^,^^4^ Therefore, to satisfy their religious obligations when receiving healthcare, Muslim patients must explain what their healthcare needs are following their religion and either negotiate with the local healthcare providers to tailor to their needs which is not provided as de facto standards, or compromise in each circumstance.^5^^,^^6^

The healthcare experiences of female Muslims living overseas have been mostly reported in the UK, Canada, and the US.^2^, ^3^, ^4^^,^^7^, ^8^, ^9^, ^10^ In addition, female Muslim women's healthcare needs are partly discussed in the studies conducted amongst refugees in South Korea,^11^ and male and female Muslims living in Japan.^5^ A qualitative study focusing on prenatal care experiences of Muslim women in Canada reported their perceived importance of being able to choose female healthcare providers and to keep their modesty throughout their pregnancy, birth, and postnatal periods.^12^ Participating Muslim women in the study were satisfied by the ways Canadian healthcare providers treated them to meet their religious obligations to keep their modesty and to cover their full body parts when receiving care. Another qualitative study was conducted in the UK to focus on the viewpoints of the healthcare providers when interacting with Muslim women for maternity care that some challenges exist due to a lack of clear definitions and practical guidance related to how to respond to Muslim women's religious needs which made them feel uncertain about how best to provide effective clinical care.^7^ A qualitative study conducted to describe the Muslims women's experience of emergency departments in the US revealed that participants preferred to be treated by female clinicians in an emergency setting, specifically for gynecological treatments, but not for any other types of treatments as it was considered as an exigency situation.^4^ To date, the perceptions, and experiences of foreign Muslim women in Japan are not well studied. Therefore, this study aims to describe the experiences of foreign Muslim women when receiving healthcare at clinics and hospitals in Japan.

## Materials and methods

A qualitative study was conducted with foreign Muslim women living in Japan, using semi-structured interviews. Phenomenological analysis was used to obtain an in-depth understanding of the participants’ perceptions and experiences from their subjective points of view.^13^ The research questions of this study are how foreign Muslim women living in Japan experience healthcare and what sociocultural challenges they face when interacting with Japanese healthcare providers. This study was part of a larger qualitative study to examine the healthcare experiences of foreign Muslims living in Japan.^5^ To answer the aforementioned research questions and to identify influencing factors on the healthcare-seeking behavior of foreign Muslims living in Japan, we interviewed them on wider issues related to their attitude, subjective norm, and perceived behavioral control regarding their healthcare needs, from their own point of views.

### Participants and recruitment

The participants were foreign Muslim women living in Japan who were from Indonesia and Malaysia and were living in the Kansai area of Japan at the time of the study. The nationalities of the participants were chosen because these are the two countries in Southeast Asia in which Muslims form the majority population, and there are a substantial number of people from these countries who are immigrating to Japan. The recruitment was carried out via gatekeepers by contacting several Muslim religious groups in the Kansai area, which were asked for their cooperation to make announcements about this research to their members. Those who were interested in taking part in this research contacted the researchers and interview appointments were made. The interviews were conducted from August 2020 to February 2021.

### Data collection and analysis methods

Interviews were conducted by the three research assistants (two Indonesians and a Malaysian) who are trained to conduct interviews, in their respective local languages. The interviews were recorded with prior permission obtained from the participants. An interview guide was developed and used during the interviews to facilitate asking questions in accordance with the research aim. The interview guide is available as a supplementary document in the previous study.^5^ Additional questions were asked to probe what the participants said. The average interview time was 66 minutes. Verbatim transcripts were made by the aforementioned three research assistants, initially in either of their respective local languages, Indonesian or Malaysian, and subsequently, English translations were created for analysis. Translations were conducted by the same research assistants (two Indonesian and one Malaysian) who were fluent in English. The data were analyzed using phenomenology.^13^ The following seven interpretative stages of phenomenology are used to guide the analytical processes; (1) acquiring a sense of each transcript, (2) extracting significant statements, (3) formulation of meanings, (4) organizing formulated meanings into clusters of themes, (5) exhaustively describing the investigated phenomenon, (6) describing the fundamental structure of the phenomenon, and (7) returning to the participants. Coding was conducted by a primary researcher (AK) and shared with the coresearchers (MD, DK, ID, HA, and TN) to refine them. A series of discussions were made over the course of the analysis process to resolve any discrepancies that arose during the analysis and finally agreed on a set of codes, categories, and themes. This study follows the standards for reporting qualitative research: a synthesis of recommendations.^14^

### Theoretical framework

**Figure 1:**
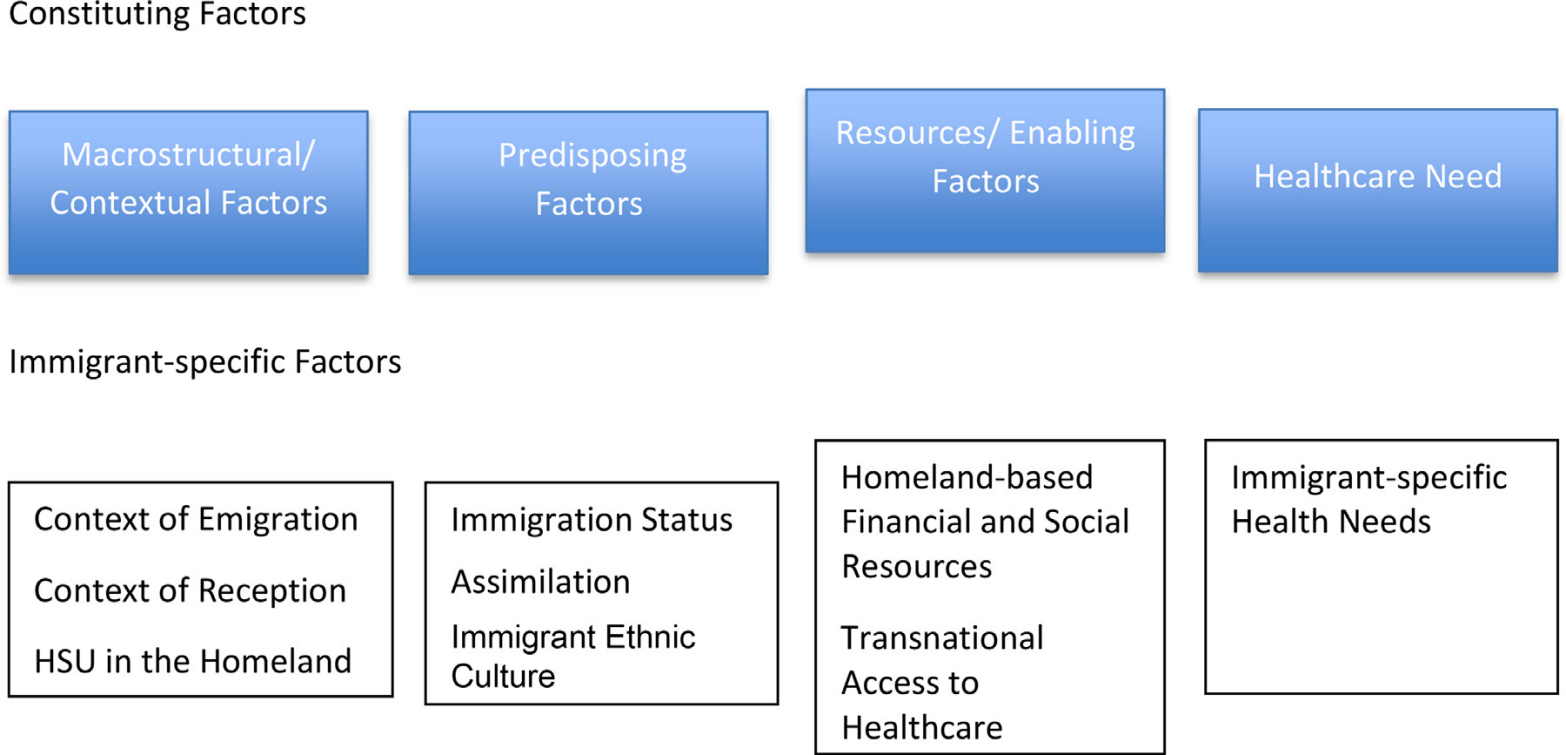
Constituting elements of the theoretical framework for immigrant health service utilization

We employed Yang and Hwang's^15^ theoretical framework on immigrant health service utilization as a framework for analysis of this study. This theoretical framework specifically addresses the disparities of health service utilization among immigrants by focusing on four factors: macrostructural factors, predisposing factors, resources, and health care needs. Figure 1 shows the constituting elements of the theoretical framework for immigrant health service utilization and the immigrant-specific factors. This was broadly adapted to capture the comprehensive view of the findings of this study, and the framework was modified to fit the unique characteristics of this study.

Kohno. Balancing religious obligations and cultural integration. AJOG Glob Rep 2025.

### Ethical considerations

Before the interview began, each participant received a detailed explanation from the interviewer in the form of a written participant information sheet as well as an oral explanation about the study, including purpose, expected risks and benefits, and anonymity of information obtained from this study. They were informed that they could withdraw from the study at any time, without informing the reason. All participants gave informed consent to be part of this study before the interviews and did not withdraw. This study was approved by the Kyoto University Graduate School and Faculty of Medicine Ethics Committee, R2516.

## Results

### Demographic characteristics of respondents

In this study, we interviewed 28 foreign Muslim women living in Japan. The overview of the participants’ characteristics is shown in Table 1. Three categories were revealed as the participants experienced healthcare in Japan: (1) meeting religious obligations as a female Muslim, (2) pregnancy and childbirth-related experiences, and (3) experiences of accessing healthcare in Japan. Each of them is elaborated as the phenomenological description of the experiences of foreign Muslim women in Japan. Table 2 shows the themes and categories.

**Table 1:** Overview of participants’ characteristics (*n*=28)

Characteristics	Number	Characteristics	Number
Nationalities Indonesian Malaysian	21 7	Occupations Students Housewife Full-time employees Part-time employees	8 10 7 3
Age in years 19–29 30–39 40–49 Fifty and above	10 14 2 2	Years living in Japan Less than 1 y 1–3 y 3–5 y More than 5 y	0 9 8 11

**Table 2:** Themes and categories

Themes	Categories
Meeting religious obligations as a female Muslim	1) Preference to be examined by female healthcare providers
	2) Keeping the Aurat (Humility in showing the opposite sex certain body parts)
	3) Conditions when receiving gynecological care
Pregnancy and childbirth-related experiences	1) Interaction with Japanese healthcare providers during pregnancy and giving birth
	2) Particular concern for halal formula milk in Japan
	3) Performing Adzan and Tahnik for the newborn baby
	4) Miscarriage experience in Japan
Experiences of accessing healthcare in Japan	1) Feeling of acceptance as a Muslim in Japan
	2) Being afraid of making too many requests to the healthcare providers to satisfy her needs as a Muslim
	3) Experiencing cultural differences of healthcare

### Meeting religious obligations as a female Muslim

This category consists of three codes; (1) preference to be examined by female healthcare providers, (2) keeping the Aurat (Humility in showing the opposite sex certain body parts), and (3) conditions when receiving gynecological care.

Most of the participating women stated their preference to be examined by a female medical doctor. Although it is their high priority, if a female doctor is not available, they said they can also be examined by a male doctor.

*If there is a female doctor, yes, go to female doctors. If there is no female doctor, then it's fine.* (No. 1, Indonesian, Student)

A typical procedure would be that the participating Muslim women will call the clinic or hospital in advance to make appointments with a female doctor, to ensure that she will be examined by a female doctor. Others checked information on the hospital or clinic websites to determine the gender of the available doctors at the facility where they plan to go. Many of the participating women stated that it is their responsibility to meet their religious obligations as female Muslims to proactively seek information to be examined by a female doctor.

*Just click one by one (websites), phone call one by one, do they have a female doctor? Well, that's how I can find Ob-Gyn, which is now my regular Ob-Gyn.* (No. 2, Indonesian, Housewife)

Regarding keeping the Aurat (humility in showing the opposite sex certain body parts), in general, Muslim women are obligated to cover their body parts other than the face and both hands from males. This is no exception when visiting clinics and hospitals in Japan. Muslim women were anxious to find ways to keep the Aurat, especially with respect to giving birth in Japan,

*I followed their rules (as what the Japanese doctor said). Because I feel it's an emergency condition with which I can't interfere. But I say that as much as possible there is no male. So, for example, when during the delivery procedure, I take off my hijab (veil) because I can't stand it… it's feeling hot. But when taken, for example, from the waiting room to the labour room, I asked to wear a hijab when I was pushed there.* (No. 2, Indonesian, Housewife)

Another problematic case was reported at the time of receiving an X-ray examination. In Japan, many of the laboratory technicians are male, and it is necessary to take off clothes when taking an X-ray. Some managed to negotiate with the technician in charge to undress only in front of the X-ray machine.

*For example, when I took an X-ray with a male nurse, I had to take off my top shirt. Then I communicated to compromise only to undress when in front of the X-ray machine only, and I was allowed to do that.* (No. 11, Indonesian, Housewife)

In some cases, the patients had to cancel the doctor's appointment, as the participating Muslim women feared exposing themselves in front of male healthcare providers when receiving medical treatment.

*Aurat problems. I will feel uncomfortable if I have to show some private parts of my body. I once had back pain, but because I was afraid that I would be asked to take off my clothes, I cancelled to see the doctor.* (No. 12, Indonesian, Student)

An experience of receiving surprising reactions from a Japanese doctor was explained by a participating foreign Muslim woman who was willing to open her hijab when examined by a male doctor, to allow him to examine her respiratory health. For Japanese doctors, as they usually find it difficult to perform checkups with Muslim women patients, it is perplexing why some Muslim women allow this while others insist firmly not to.

*Most of the hospitals here in Osaka have some knowledge about Muslim. For example, during respiratory check, a doctor might say, “Can you open your head scarf a bit?” When I gave permission, most doctors are surprised because they said other patients, especially from Pakistan or India does not consent.* (No. 28, Malaysian, Housewife)

### Pregnancy and childbirth-related experiences

Many of the participants experienced pregnancy and childbirth while living in Japan. There were some specific worries and concerns for them as foreign Muslim women when getting pregnant and giving birth in Japanese clinics and hospitals. The categories include (1) interaction with Japanese healthcare providers during pregnancy and giving birth, (2) particular concern for halal formula milk in Japan, (3) performing Adzan and Tahnik for the newborn baby, and (4) miscarriage experience in Japan.

When interacting with Japanese healthcare providers, foreign Muslim women living in Japan needed to discuss and negotiate with them to fulfill their needs as Muslims to go through childbirth in compliance with Muslim obligations on several points. One such notable experience was with regard to bringing back home the placenta for burial. The story of one participant described how they needed to make special efforts to persuade by explaining to a doctor on multiple occasions and waiting for them while they took time to consider and coordinate.

*We told the doctors that we have to take the placenta back home, and they said no initially, since it's a procedure in Japan for it to be disposed of (in the hospital). Then we told them it was an Islamic procedure and we wanted to bring it home, and we told them that we won't be using it for anything against the law. After consideration, we were told that we can bring it home on the condition that they will need to freeze it first.* (No. 28, Malaysian, Housewife)

There were also recurring issues of wanting to be dealt with female doctors, provision of halal food, and being allowed to perform prayers during hospitalization were stated as what the participants wanted while receiving care at Japanese clinics and hospitals for childbirth. They needed to explain why and what they needed and seek for permission from Japanese healthcare providers beforehand.

*It was difficult to explain our needs for a prayer space, especially during relative visitation. But I was lucky as the doctor was quite understanding and said, “In your case we will prepare seafood and vegetables and treatment will be handled by female doctors.” Alhamdulillah (thank God) there was no problem onwards.* (No. 27, Malaysian, Full-time Employee)

As part of their efforts to keep the Aurat, participants were keen to find out whether they would be allowed to wear hijab during the delivery process. Some hospitals in Japan forbid Muslim women to wear hijab during childbirth for the reason of securing safe and hygienic conditions to provide medical care, while others allow them. This is the experience of a woman who experienced multiple childbirths at different hospitals in Japan experienced both cases.

*In the contraction or waiting room, I still wore my hijab. However, when I was about to give birth, the doctor advised me to take off my hijab for fear of interfering with the birth process. Giving birth is a matter of life and death, right? So, I took off my hijab.* (No. 20, Indonesian, Housewife)

There was a particular concern when giving halal formula milk to their newborn child in Japan. In Japan, it is quite difficult and costly to access halal formula milk. It is simply not available at regular stores, so it must be bought at special places or imported from overseas. A mother had to take time and effort in searching for information and contacting the formula milk production company to know the ingredients of the formula milk in Japan.

*There is a component called taurine (in the formula milk). It turned out that after I crosschecked the company, everything was made from animals. So, what has been recommended by those who like to check halal it turned out are haram, especially those that they recommended.* (No. 2, Indonesian, Housewife)

Some of the participating women experienced performing Adzan (Islamic prayer) and Tahnik (Islamic ceremony of rubbing the palate of a newborn baby with honey, sweet juice, or pressed dates) for a newborn baby soon after the baby was born, and some communications were done between the couple and the Japanese healthcare providers to obtain prior permission, so that the event will not be perceived by the Japanese healthcare providers who are not familiar with why or how such events will take place, to be a strange experience.

*Because my husband could not accompany me during childbirth, he managed to give adzan when he visited my room. I asked the doctor to give us a moment with the baby. We can request it on the form when we first admitted to the hospital and usually, they will understand.* (No. 9, Indonesian, Housewife)

Some of the participants had to go through a miscarriage experience while living in Japan. As it is important that the fetus after 8 weeks should be buried instead of cremated, the couple searched for information regarding burial in Japan, which is difficult and could be at a faraway place depending on where they live.

*Last time, there was a friend of a friend, whose wife had a miscarriage … but the miscarriage was still small, so in Islam, this foetus's condition has not yet been blown by the spirit (In Islam, the foetus is believed to become a living soul after 120 days gestation). Even though it doesn't have to be buried, because the foetus's life hasn't been blown yet, so it can be buried anywhere. But it was forbidden by the hospital and by the city municipal government! So, that's why this person suddenly looked for a Muslim burial place… In the end, the foetus was cremated as far as I know.* (No. 2, Indonesian, Housewife)

### Experiences of accessing healthcare in Japan

Participants had varied experiences when accessing healthcare in Japan. This theme constitutes the categories of (1) feeling of acceptance as a Muslim in Japan, (2) being afraid of making too many requests to the healthcare providers to satisfy her needs as a Muslim, and (3) experiencing cultural differences in healthcare.

Some of the participants, when experiencing healthcare in Japan, had experienced the feeling of being accepted as a Muslim patient. In so doing, some of them felt the need to become reserved so as not to burden the Japanese healthcare providers too much.

*In A city (middle-sized city in Japan), there are already many foreigners living there, so Japanese are used to seeing foreigners, I stayed in the room with other patients. But so far, there are no problems in the acceptance of Muslims among other patients there, from my own experience.* (No. 20, Indonesian, Housewife)

Also, some participants raised their concerns regarding their worries about making too many requests to the healthcare providers to satisfy her needs as Muslim patients in Japan.

*I am afraid they (Japanese healthcare providers) would think that I request too many terms and conditions as a Muslim. If I tell them that I can't consume alcohol, gelatine, or pork derivatives, then they will get confused on how to treat me. So, it has been my lengthy battle, which one do I need to do first, healing my body or following Islamic teachings? In the end, I never asked the doctors because I was afraid of their responses.* (No. 14, Indonesian, Full-time Employee)

Regarding the experience of cultural differences in healthcare, some participants compared how they can access healthcare in their home countries of Indonesia and Malaysia, with the healthcare access in Japan.

*It's a bit different. In Indonesia, I use BPJS (Indonesia's Health Insurance System). If I want to get medical treatment, the BPJS members have a different level of health facilities starting from level 1 or primary care, for example, having to go to the PUSKESMAS (Health Community Center) or doctor's clinic appointed by the BPJS. From that level 1 service, we can only go to the hospital if we are given a referral. Meanwhile in Japan, even though there are referrals, you can go directly to the hospital, for example to a tertiary hospital, without having to go to the clinic first. You can go directly to a rather large hospital.* (No. 21, Indonesian, Housewife)

**Figure 2:**
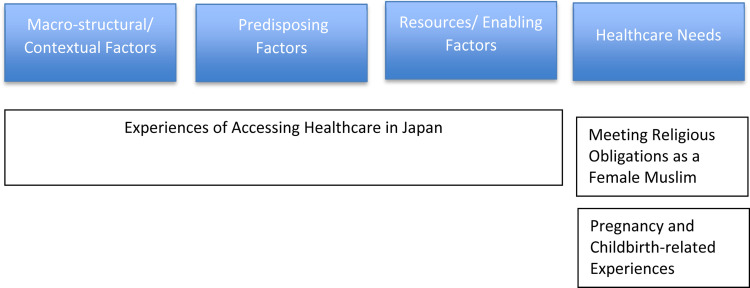
Alignment of the three themes with the theoretical framework on immigrant health service utilization

Finally, the resulting three themes are shown in alignment with the theoretical framework of Yang and Hwang on immigrant health service utilization. Figure 2 shows the alignment in graphical form. The theme of “Experience of Accessing Healthcare in Japan” encompassed the elements of macrostructural/contextual, predisposing, and resources/enabling factors. As Muslim women in Japan were comparing their homeland healthcare experience with that of Japan, as summarized in the category “Experiencing cultural differences of healthcare,” they were bound by the macro-structural/contextual factors. Predisposing factors were evident in almost all aspects of what the participants told through their narratives. They were assimilating to the Japanese healthcare system as can be found in such category as “Being afraid of making too many requests to the healthcare providers to satisfy her needs as a Muslim.” At the same time, there were some enabling factors such as a “Feeling of acceptance as a Muslim in Japan.” Most of the Muslim women's efforts to meet their religious obligations are the aspects of achieving their healthcare needs. The two themes of “Meeting Religious Obligations as a Female Muslim” and “Pregnancy and Childbirth-related Experiences” are mainly regarding their healthcare needs.

Kohno. Balancing religious obligations and cultural integration. AJOG Glob Rep 2025.

## Discussion

This study revealed three salient issues for foreign Muslim women as they experienced healthcare in Japan. In the discussion section, the identified issues are elaborated and compared with insights gained from previous studies dealing with similar cases of how healthcare was experienced by migrant Muslim women in other non-Muslim countries’ healthcare contexts. Additionally, recommendations are proposed to enhance the capacity of Japanese healthcare providers in examining female Muslim patients.

Firstly, there were some challenging issues for the female Muslim patients in this study when they tried to meet their religious obligations. Regarding the issue of gender-concordant care, they were trying their best to find out beforehand to make sure to be examined by a female doctor. Such efforts were made by calling the clinics and hospitals beforehand and checking their websites. However, in some cases, due to unavoidable circumstances, some of the female Muslim women accepted to receive healthcare from a male doctor. It has been known through a study conducted in the US that modest Muslim women tend to delay their care when gender-concordance care is not available. Another literature review that investigated the Muslim women's barriers in healthcare utilization highlighted that the intersection of religion, culture, and gender for Muslim women has unique implications for their healthcare utilization.

In many cases, Japanese healthcare providers have no prior understanding of the religious and cultural needs of Muslim women. As was highlighted in a study of the healthcare experience of Iraqi Muslim women in the US, the problem was that there was a gap between what the healthcare providers thought as de facto procedures in healthcare provision, and the conflicting values and expectations held by Muslim women.^9^ The concept of patient-centered care for Muslim women is critical in this context.^10^ As a recommendation to achieve patient-centered care for Muslim women, conducting educational training for healthcare providers to enhance understanding of basic religious and cultural beliefs of Muslim patients and provision of hints to facilitate improving patient-provider relationships are vital.

Secondly, while they experience pregnancies and childbirths in Japan, some critical issues were identified that are related to their religion. Generally, female Muslim patients in Japan interact with Japanese healthcare providers by discussing, negotiating, and communicating patiently during their pregnancy and giving birth. In addition, there were some specific issues of not being able to obtain halal formula milk in Japan, and their wishes for performing Adzan and Tahnik for the newborn baby were voiced by the participants. On these points of interaction between female Muslim patients and healthcare providers, a qualitative study to investigate the immigrant Muslim women's maternity care needs and barriers in the United States revealed that the women “experienced discrimination, insensitivity, and lack of knowledge about their religious and cultural practices.^12^” In this study, although the voices heard from the participating Muslim women in Japan were generally not negative, they had some issues with respect to their religious obligations such as bringing back home the placenta for burial, which required careful discussion and negotiation with the Japanese healthcare providers. Reitmanova et al highlighted that lack of knowledge on the part of healthcare providers, it may lead to an inability for the healthcare providers to “provide knowledgeable health guidance and maternity information that takes women's religious needs into consideration.^3^^,^^16^” Therefore, it is vital to empower healthcare providers with information about Muslim women's religious needs and concerns.

Regarding the unavailability of halal formula milk in Japan, there is no previous study focusing on this topic. Although it is a critical issue for Muslim mothers who gave birth in Japan and cannot produce breast milk, simple solutions are not provided. Two perspectives are important for further consideration in this context. One is how to relieve the anxiety and nervousness of a Muslim mother for not being able to provide halal formula milk to her newborn baby. On this point, a standardized global religious guideline for those Muslims who are giving birth in non-Muslim countries may help alleviate such anxiety. Another perspective is regarding the awareness-building of midwives and nurses in clinics and hospitals in Japan regarding halal formula milk. As Japanese healthcare providers may perceive the case as child neglect if a Muslim mother refuses to feed a baby with non-halal formula milk, which may be interpreted as a mother not willing to provide sufficient nutrition to a newborn baby, the information must be shared widely amongst Japanese healthcare providers so that the miscommunication will not arise.

Regarding the third theme of “experiences of healthcare access in Japan,” there was a mix of positive and negative experiences of female Muslims in Japan. On the positive side, the participants felt being accepted as they communicated with Japanese healthcare providers about their religious needs and obligations while receiving healthcare. When they stated their requests clearly to the Japanese healthcare providers, on such issues as gender-concordance care, halal medication, and foods, Japanese healthcare providers were cooperative in making efforts to accommodate such requests as much as possible. Previous studies highlighted the importance of a collaborative patient-provider relationship that is based on flexibility and giving female Muslim patients to speak up about their religious beliefs so that they do not have to endure nor give up their religious values^10^ as immigrants in different cultural and religious contexts. At the same time, we observed in this study that some of the foreign Muslim women voluntarily refrained from informing all their religious requirements to the Japanese healthcare providers, and they were raising concerns for their future healthcare access in Japan, thinking that if they ask too much, it will burden the Japanese healthcare providers. Similar findings were reported in the previous study conducted amongst Muslim patients in the US.^2^ The study participants were characterized by Padela et al as reticent. They sometimes felt burdened when they needed to constantly educate the American healthcare providers about their healthcare needs as Muslims. As a solution, Padela et al proposed promoting dialogue between hospital administrators and minority community representatives. Such arrangements between Japanese hospital administrators and the local Muslim communities in Japan can ease the process of enhancing mutual understanding.

Based on the findings of this study, several recommendations are provided to enhance the competence of Japanese healthcare providers in delivering culturally responsive care to female Muslim patients. Notably, a training program is essential to equip healthcare providers with the necessary knowledge and insights for effective interactions. This program could be implemented as an instructional video illustrating common requests made by female Muslim patients in clinical and hospital settings, along with exemplary responses from healthcare providers. Furthermore, policy reforms within the Japanese healthcare system are necessary to establish better incentives for hospitals and clinics that accommodate foreign patients, particularly those from diverse cultural and religious backgrounds, including Muslims. Under the current reimbursement framework, medical institutions can charge additional fees for interpretation services when examining non-Japanese-speaking patients. However, there is no system in place that allows healthcare providers to claim additional fees for delivering culturally sensitive care to foreign patients. Implementing a new reimbursement scheme for culturally sensitive healthcare services would benefit both Japanese healthcare providers and foreign patients, creating a mutually advantageous solution.

### Strengths and limitations

This study revealed the lived experiences of foreign Muslim women as they receive healthcare in Japan. To date, there is no study focusing on this topic. Therefore, the result of this study adds new insights to Japanese healthcare providers when interacting with foreign Muslims living in Japan. This study has two limitations. Firstly, the participant of this study is limited to Muslim women from Indonesia and Malaysia. The experiences and perceptions of Muslim women from other regions, such as the Middle East and Africa, may differ from what was found in this study, due to variations in cultural norms, interpretation of Islamic modesty, and the actual healthcare services provided in the respective countries. Secondly, the participants of this study are mainly students, full-time workers, and accompanying family members. We were able to recruit some but limited numbers of Indonesian technical trainees in this study. Since Muslim women from other countries such as the Middle East and African countries, or different occupation/residence types may have different interpretations of Islam, the results of this study may not apply to all Muslim women in Japan.

## Conclusion

This study described the healthcare experiences of foreign Muslim women in Japan as a phenomenological qualitative study. The migrating Muslim women encounter challenges in meeting their religious obligations when receiving healthcare in Japan. The findings of this study may add insights to healthcare providers regarding how to treat foreign Muslim patients in providing culturally and religiously sensitive and compassionate care. Sharing information amongst healthcare providers about what and why foreign Muslims are obligated about certain points while receiving healthcare and preparation of a set of easy-to-implement solutions will ease the process for both foreign Muslim women and healthcare providers.

## CRediT authorship contribution statement

**Ayako Kohno:** Writing – review & editing, Writing – original draft, Software, Resources, Project administration, Methodology, Investigation, Funding acquisition, Formal analysis, Data curation, Conceptualization. **Maznah Dahlui:** Writing – review & editing, Formal analysis. **Inge Dhamanti:** Writing – review & editing, Formal analysis. **David Koh:** Writing – review & editing, Formal analysis. **Hanif Abdul Rahman:** Writing – review & editing, Formal analysis. **Takeo Nakayama:** Writing – review & editing, Supervision, Formal analysis.
